# Artesunate inhibits epithelial-mesenchymal transition in non-small-cell lung cancer (NSCLC) cells by down-regulating the expression of *BTBD7*

**DOI:** 10.1080/21655979.2020.1834727

**Published:** 2020-10-27

**Authors:** Jing-Si Wang, Ming-Juan Wang, Xiao Lu, Jiao Zhang, Quan-Xing Liu, Dong Zhou, Ji-Gang Dai, Hong Zheng

**Affiliations:** a Department of Thoracic Surgery, Xinqiao Hospital, Army (Third) Military Medical University, Chongqing, China; b Department of Anesthesiology, Chonggang General Hospital, Chongqing, China

**Keywords:** Non-small-cell lung cancer (NSCLC), EMT, artesunate, *BTBD7*

## Abstract

In recent years, more and more studies have shown that antiparasitic drugs can affect a variety of biological processes of tumor cells and exhibit a potential anti-tumor activity. Although artesunate (ART), a strong bioactive derivative of artemisinin and widely used clinically against malaria, was found to have an inhibitory effect on tumor cells, it is still unclear whether ART could regulate the tumor malignancy of non-small-cell lung cancer (NSCLC) cells. In this study, we aimed to investigate the effect of ART on migration capacities in NSCLC cell lines of A549 and H1975. Cell migration capacity was remarkably inhibited by ART treatment. The expression of epithelial marker E-cadherin was upregulated, while mesenchymal markers (N-cadherin, vimentin and FN1) were inhibited by ART in both protein and mRNA levels in A549 and H1975 cells, indicating ART could suppress the epidermal interstitial transformation (EMT) of NSCLC cells. Meanwhile, *BTBD7* was found highly expressed in tumor tissues of NSCLC patient and associated with poor prognosis. The anti-migration activity of ART was found to be mediated by the inhibition of *BTBD7* mRNA expression and was reversed when the cells were transiently transfected with the *BTBD7* overexpression plasmid. Our study demonstrated the potent anti-migratory activity of ART, thereby presenting it as a new candidate for clinical therapy in NSCLC.

## Graphical abstract

**Figure:**
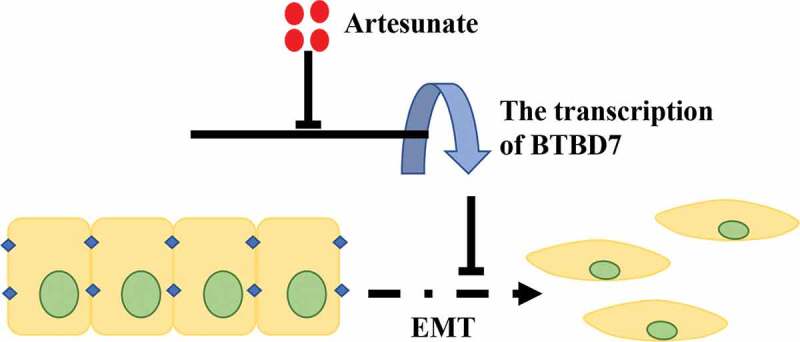

## Introduction

Lung cancer is the most common malignant tumor and is classified into non-small cell lung cancer (NSCLC), which accounts for 85% of all lung cancer cases, and small cell lung cancer (SCLC), which accounts for the remaining 15% [1,2]. Lung cancer has the highest incidence and mortality among all cancers and accounts for approximately 1.6 million deaths each year, making it the leading cause of cancer-related death around the world [3]. Although great progress has been made in the current treatment strategies, including surgery, radiotherapy, and chemotherapy, the underlying mechanism of lung cancer is still unclear, and its 5-year survival rate for all people with all types of lung cancer is only 19% [4]. Moreover, most patients are diagnosed at an advanced stage, with metastasis, which results in a poor survival rate [5].

Epithelial-mesenchymal transition (EMT) is a biological process involving the phenotypic transformation of epithelial cells to mesenchymal cells under specific physiological or pathological conditions [6,7]. Cells undergoing EMT are characterized by cell-polarity loss and the acquisition of the function of cytoskeleton remodeling [8]. Accumulating evidence indicates that EMT plays a key role in the invasion and metastasis of breast cancer [9], lung cancer [10], colorectal cancer, as well as other cancers [11]. EMT is also characterized by a decrease in the expression of epithelial proteins, including E-cadherin, and an increase in the expression of mesenchymal proteins, such as vimentin [12]. The decrease in the expression of E-cadherin is closely related to the malignancy of NSCLC, and a positive correlation exists between the expression of vimentin and pathological grade of NSCLC [13,14].

Currently, artemisinin and its combination therapies are considered standard treatments against malaria [15]. Artesunate (ART), a principal derivative of artemisinin, is a strong bioactive compound that is widely used in anti-malaria drugs [16,17]. Recent studies have shown ART exhibits an anti-tumor capacity by exerting its inhibitory effect on tumor cell migration, proliferation, and cell cycle arrest. ART has mainly been found to affect the cytoskeleton structure, chromosomal stability, and cell migration ability [18,19]. In this study, we aimed to investigate the anti-tumor activity of ART in the lung adenocarcinoma cell lines, A549 and H1975, and elucidate the underlying mechanism, which could provide new perspectives for clinical cancer therapy.

## Materials and methods

### Cell lines

Human NSCLC cells H1975 and A549 used in this study were purchased from the Library of Typical Culture of the Chinese Academy of Sciences (Shanghai, China), and then cultured in DMEM (Hyclone, USA) containing 10% FBS (Gibco, USA). The cells were incubated in a 37°C constant temperature and humidity chamber. The cells were subcultured every 2–3 days and used at the logarithmic phase throughout the study. ART (MCE, USA) was dissolved in dimethyl sulfoxide (DMSO, Sigma-Aldrich, USA) and stored in the dark. The final dilution of DMSO was less than 0.2%.

### Western blotting

The cells were treated with the indicated concentration of ART (12.5, 25 and 50 μg/ml) for 24 h, and then were lysed with RIPA lysis buffer (Beyotime, China). The total protein concentration of lysates was determined by BCA kit (Beyotime, China). The proteins were separated on 10% SDS-PAGE gels and then wet-transferred onto PVDF membranes. Then the membranes were blocked with 5% BSA buffer followed by incubation with rabbit polyclonal anti-human E-cadherin, N-cadherin, fibronectin 1 (FN1), vimentin, anti-β-actin, and *BTBD7* antibodies overnight at 4°C (all antibodies were purchased from Abcam, UK, and used at a 1:1000 dilution). All the membranes were subsequently incubated with secondary antibodies for 2 h at room temperature. The relative grayscale values were measured by using the ImageJ software 1.51d (National Institutes of Health, USA).

### qRT-PCR

1 × 10^6^ NSCLC cells were inoculated in 6-well plates. After treated with ART (50 µg/mL) for 24 h, cells were harvested using TRIzol (Invitrogen, USA), and total RNA was extracted. Reverse transcription was performed using the PrimeScript™ RT reagent kit with gDNA Eraser (TaKaRa, Japan). The mRNA level of *BTBD7* and EMT markers was detected by SYBR quantitative PCR assay with TB Green™ Premix Ex Taq™ II (Tli RNaseH Plus) (TAKARA). The primer sequences used for detecting *BTBD7* transcriptional level were: forward primer: 5′-TGTATACCGACGTG GTGGACCTC-3′, reverse primer: 5′-CTGCGACGAGAGCCTGAACTTC-3′. The primer sequences used for detecting FN1 transcriptional level were: forward primer: 5′- ATGCAACGATCAGGACACAAGGAC-3′, reverse primer: 5′- TGCCTCTCAC ACTTCCACTCTCC-3′. The primer sequences used for detecting E-cadherin (CDH1) transcriptional level were: forward primer: 5′- GCTCTTCCAGGAACCTCTGTGATG-3′, reverse primer: 5′- TGTAAGCGATGGCGGCATTGTAG-3′. The primer sequences used for detecting N-cadherin (CDH2) transcriptional level were: forward primer: 5′- AGGCGTCTGTAGAGGCTTCTGG-3′, reverse primer: 5′- GAGGCTGTCCTTC ATGCACATCC-3′. The primer sequences used for detecting vimentin transcriptional level were: forward primer: 5′- GACGCCATCAACACCGAGTT-3′, reverse primer: 5′- CTTTGTCGTTGGTTAGCTGGT-3′. The internal reference adopted was GAPDH (Sangon Biotech, China).

### Wound healing assay

A549 and H1975 cells were seeded in 6-well culture plates at a concentration of 2.5 × 10^5^ cells/ml and incubated overnight. After aspirating the medium, a pipette tip (1000 µL) was used to create a straight uniform linear scratch across center of each cell monolayer, followed by gently washing with PBS to remove cellular debris. Cell wound healing images were taken at 0 h and 24 h following ART treatment. The relative wound width was analyzed using the ImageJ software.

### Transwell assay

Another assay for detecting the migrated suppression ability of ART in NSCLC cell lines was transwell. First, 4 × 10^4^ cells were seeded into the upper transwell chamber (Millipore, Germany) in 200 µL FBS-free medium; then, 500 µL cell medium plus 10% FBS was added to 24-well plate. Both the upper and lower chambers were treated with 50 µg/mL ART for 24 h. The non-migrated cells were lightly removed and the remaining cells were fixed with 4% paraformaldehyde and then stained with 0.1% crystal violet. Cells in twenty-five visual fields were imaged and counted per experiment.

### BTBD7 overexpression assay

To further investigate the effect of *BTBD7* in the migration inhibition effect of ART, we constructed *BTBD7*-overexpressed plasmid and control plasmid in Sangon Biotech (Shanghai, China). *BTBD7* coding sequence (GenBank: BC047071.1) was cloned into pcDNA3.1(+) and followed with CMV promoter. The cells were seeded in plates and divided into four groups as follows: control, OVE-*BTBD7*, ART, and OVE-BTBD plus ART. A549 and H1975 cells were transfected with the control and *BTBD7*-overexpression plasmids using the Lipofectamine 3000 transfection (Invitrogen, USA) for 12 h, and the cell culture was replaced. Then, cells were treated with ART or medium for 24 h. For detecting the influence of *BTBD7* overexpressing on cell migrated capacity, transwell, wound healing assay and western blot were performed.

### Clinical samples

The tissue microarray including 87 paired tumor tissues and corresponding non-cancerous tissue specimens were obtained from Lung adenocarcinoma patients diagnosed and operated at the Xinqiao Hospital, the Third Military Medical University, from 2008 to 2013. Histochemical staining and scoring were proceeded as described previously [20].

### Immunohistochemistry and scoring

*BTBD7* immunohistochemistry was conducted on formalin-fixed and paraffin-embedded tissue sections (5-μm thick). The slides were stained by labeled streptavidin-biotinylated peroxidase method (ZSGB-BIO, Beijing, China) as per the manufacturer’s instructions. The sections were first incubated with primary antibodies to *BTBD7* (1:25; Abcam, Camb, UK) at 4°C overnight and then incubated with specific secondary HRP-conjugated antibodies (Dako, Santa Clara, CA). The expression of *BTBD7* was detected and scored using a semi-quantitative staining index. The index was calculated by multiplying the expression extent score (0 points: <5% positive cells, 1 point: 5–25% positive cells, 2 points: 26–50% positive cells, 3 points: 51–75% positive cells, and 4 points: >75% positive cells) by the staining intensity score (0 points: negative expression, 1 point: weak expression, 2 points: moderate expression, and 3 points: strong expression). A cutoff value of 4 points was used to define high/low expression scores, and all data were analyzed using X-tile software (version 3.6.1; New Haven, CT, USA).

### Statistical analysis

Enumeration data in this study are presented as the mean ± SD. The statistical analysis between groups was performed using the SPSS 19.0 software (SPSS, Inc., Chicago, IL, USA) using one-way ANOVA method for multiple group comparison or unpaired Student’s t-test between two groups. The difference in results was statistically significant as p-value <0.05.

## Results

### ART inhibits cell migration in human NSCLC cells

**Figure 1.**
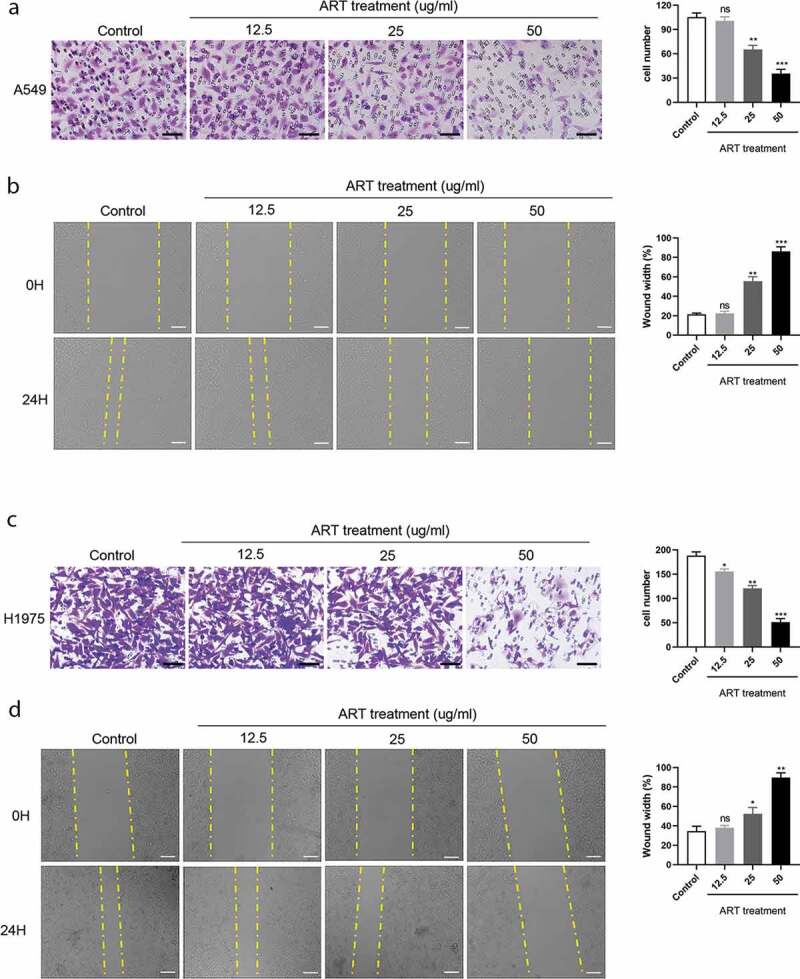
ART inhibits migration in NSCLC cells. 2 × 10^4^ A549 cells (a) and H1975 cells (c) were inoculated in transwell chamber with FBS-free medium and treated with the indicated concentration of ART; 24 h later, cells were fixed and stained. The migration capacity was measured by determining the mean number of cells observed on the lower surface of the transwell membrane in 25 visual fields, Scale bar, 50 μm; 1 × 10^6^ A549 cells (b) and H1975 cells (d) were inoculated in a 6-well plate overnight; then the cell layers were scratched with 1000 µL tips. Cells were incubated with medium containing 3% FBS and treated with the indicated concentration of ART. The scratch was photographed at 0 h and 24 h, and the relative wound width was detected using the Image J software, Scale bar, 100 μm. ns, not significant. The data were presented as mean ± standard deviation for 3 independent experiments. *p < 0.05, **p < 0.01, ***p< 0.001

To assess the effect of ART on the migration capacity of NSCLC cells, transwell and wound healing assays were performed. A549 and H1975 cells were treated with 0, 12.5, 25, and 50 µg/mL ART for 24 h, and then the cell number was analyzed. As shown in Figure 1(a and c), no significant decreased number of migrated cells was observed for A549 cells (P > 0.05), but for H1975 cells (P < 0.05) when treated with 12.5 µg/mL ART compared with untreated control. However, 25 and 50 µg/mL ART treatment showed a remarkable suppressive effect on the number of A549 and H1975 cells (P < 0.01 or P < 0.001). Consistent with the transwell assay results, the wound healing assay results showed that the cell migration capacity of A549 and H1975 cells in the ART treatment group was remarkably inhibited at the concentration of 25 and 50 µg/mL for 24 h (Figure 1(b and d), P < 0.05, P < 0.01 or P < 0.001). In conclusion, ART inhibited the migration capacity of the two NSCLC cell lines dependent on a dose manner.

### ART suppresses the occurrence of EMT

**Figure 2.**
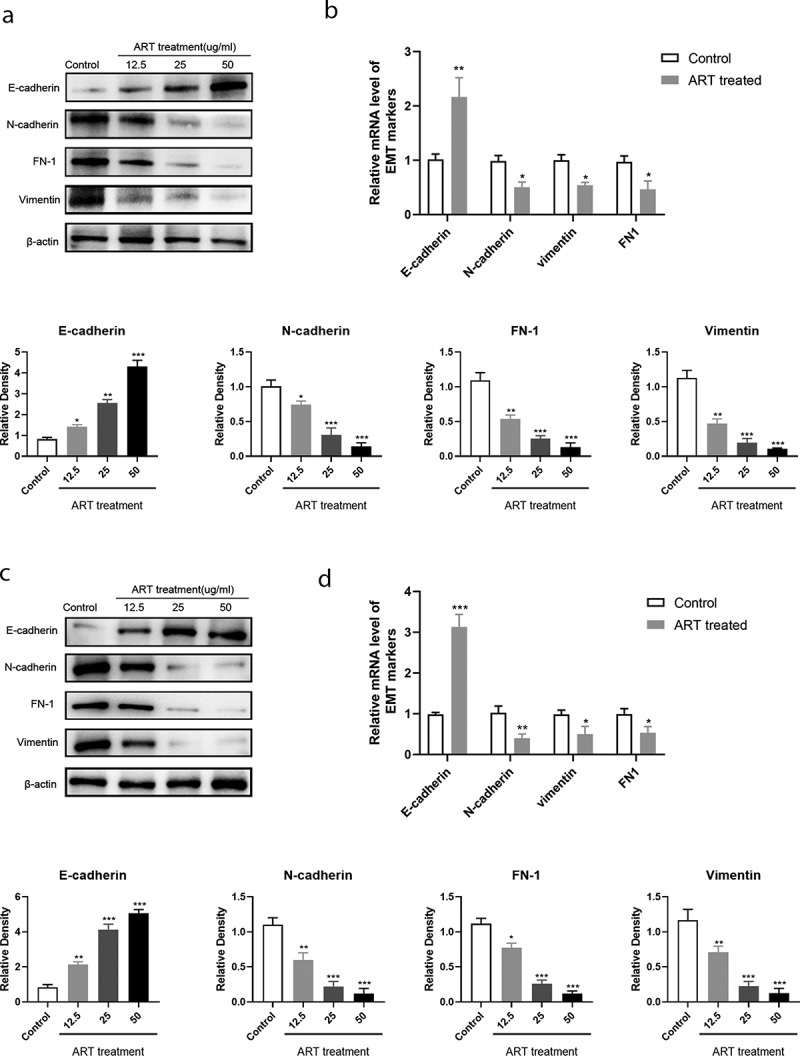
ART up-regulated the expression of E-cadherin and inhibited the expression of mesenchymal protein markers. 1 × 10^6^ A549 cells (a) and H1975 cells (c) were inoculated in a 6-well plate overnight and then treated with the indicated concentration of ART for 24 h; the level of EMT-related proteins was detected by western blotting, and the relative grayscale value was measured using Image J software. 1 × 10^6^ A549 cells (b) and H1975 cells (d) were inoculated in a 6-well plate overnight and then treated with 5ug/ml ART for 24 h; the total RNA was collected and the transcriptional level of EMT-related markers were detected with qRT-PCR. The data were presented as mean ± standard deviation for three independent experiments. *p < 0.05, **p < 0.01, ***p< 0.001

The underlying mechanism of tumor metastasis is still a research hotspot in cancer therapy. Growing evidence shows that tumor metastasis and invasion are often accompanied by the disappearance of the epithelial cell morphological characteristics and the appearance of mesenchymal phenotype characteristics. The migration ability of tumor cells undergoing EMT increases, and they become more aggressive. To investigate whether ART treatment inhibits the process of EMT in NSCLC cells, the protein and mRNA levels of E-cadherin and other EMT markers were detected in A549 and H1975 cells by western blotting. As shown in Figure 2(a and b), the expression of the epithelial cell marker (E-cadherin) was increased when cells were treated with 12.5 µg/mL ART, whereas the level of mesenchymal cell markers, including N-cadherin, vimentin, and FN1, was significantly reduced (Figure 2(c and d)). ART was found to suppress EMT in a dose-dependent manner. This result indicates that ART treatment inhibits the migration capacity of NSCLC cells by repressing EMT.

### ART suppresses the transcription of BTBD7

**Figure 3.**
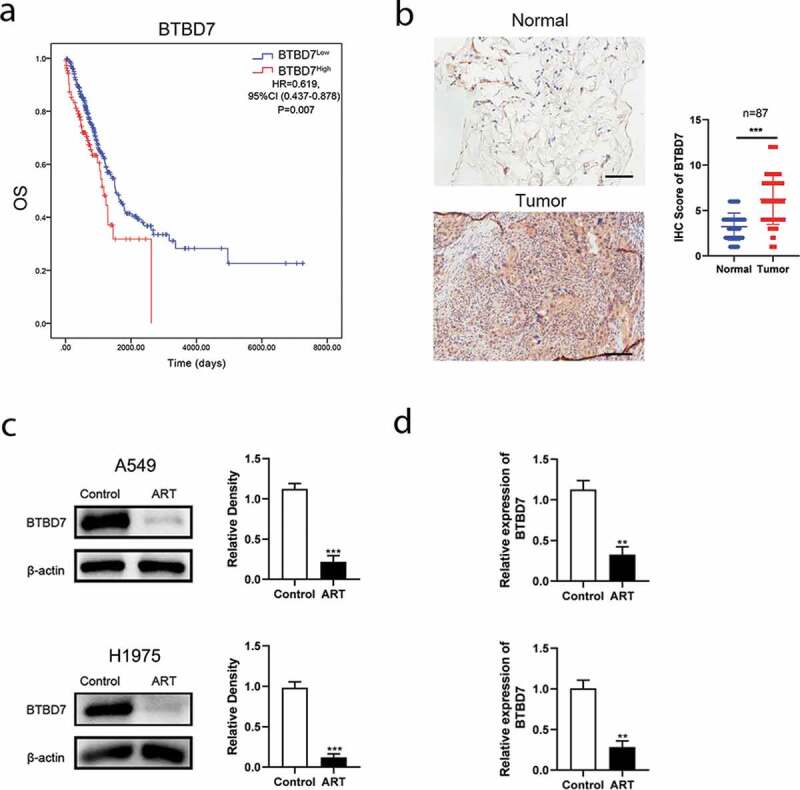
ART inhibited lung cancer cell metastasis by downregulating the transcription of *BTBD7*. (a) Overall survival analysis of NSCLC patients from TCGA database. (b) Histochemical staining and analysis in 87 paired NSCLC patients, Scale bar = 100 μm. (c) 1 × 10^6^ A549 cells (*up*) and H1975 cells (*bottom*) were inoculated in a 6-well plate overnight and then treated with 50 µg/mL ART; 24 h later, the protein level of *BTBD7* was detected by western blotting, and the relative grayscale value was measured by using Image J software; (d) after ART treatment for 24 h, the total RNA was extracted by using TRIzol and reversed transcribed in A549 (*up*) and H1975 (*bottom*) cells; *BTBD7* mRNA level was detected by RT-qPCR. The data were presented as mean ± standard deviation for 3 independent experiments. **p < 0.01, ***p< 0.001

Studies have reported that *BTBD7* was closely correlated with the process of EMT and poor prognosis in patient with NSCLC [21–23]. We next investigated whether *BTBD7* was involved in ART-induced EMT inhibition in NSCLC cells. Data from TCGA database showed that patients with high *BTBD7* expression had a poor prognosis in overall survival (Figure 3(a)), and immunohistochemical staining carried out in all 87 paired HCC sample also confirmed that *BTBD7* was highly expressed in tumor tissues of NSCLC (Figure 3(b)). Next, we detected the expression level of *BTBD7* in A549 and H1975 cells by western blotting. Interestingly, the expression of *BTBD7* decreased significantly after ART treatment (Figure 3(c)). Further, detection of the mRNA level of *BTBD7* through the qPCR assay revealed that the transcription of *BTBD7* was inhibited upon ART treatment as compared to that in the control group (Figure 3(d)), which indicates that ART treatment could downregulate the transcription of *BTBD7* in NSCLC cells.

### ART upregulates the protein level of E-cadherin and downregulates other EMT markers through BTBD7

**Figure 4.**
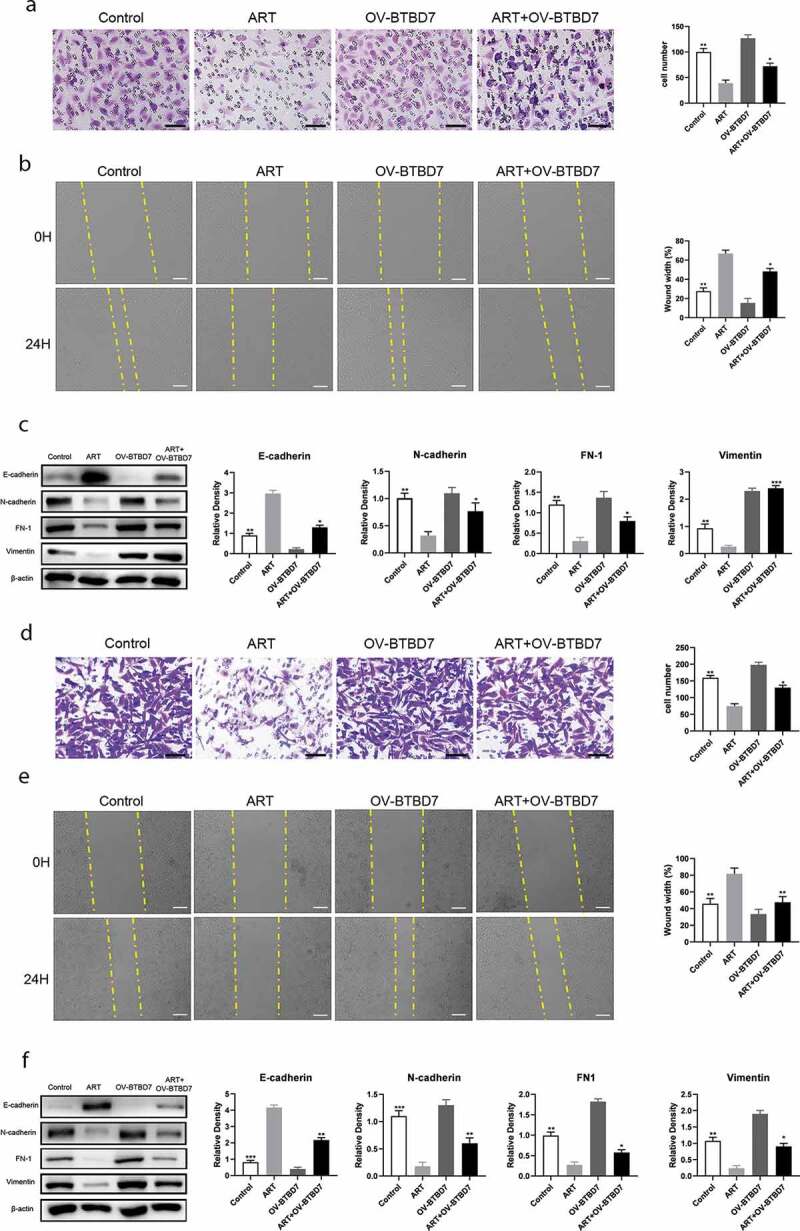
Migration inhibition mediated by ART was reversed when *BTBD7* was overexpressed in NSCLC cells. 2 × 10^4^ A549 cells (a) and H1975 cells (d) were transfected with the *BTBD7* overexpression plasmid overnight; then they were inoculated in the transwell chamber with FBS-free medium and treated with 50 µg/mL ART, 24 h later, cells were fixed and stained. The migration capacity was measured by determining the mean number of cells observed on the lower surface of the transwell membrane in 25 visual fields; Scale bar, 50 μm; 1 × 10^6^ A549 cells (b) and H1975 cells (e) were inoculated in 6-well plate and transfected with the *BTBD7* overexpression plasmid overnight; then, the cell layers were scratched with 1000 µL tips. Cells were incubated with medium containing 3% FBS and treated with 50 µg/mL ART. The scratch site was photographed at 0 h and 24 h, and the relative wound width was detected using Image J software. Scale bar, 100 μm. 1 × 10^6^ A549 cells (c) and H1975 cells (f) were inoculated in a 6-well plate and transfected with the *BTBD7* overexpression plasmid overnight; then, they were treated with 50 µg/mL ART for 24 h. The expression level of EMT-related markers was detected by western blotting, and the relative grayscale value was measured. The data were presented as mean ± standard deviation for 3 independent experiments. *p < 0.05, **p < 0.01, ***p < 0.001

To identify whether ART inhibits NSCLC cell migration through *BTBD7*, we constructed a *BTBD7*-overexpression plasmid and a corresponding control plasmid for transfection into A549 and H1975 cells. The transwell assay results showed that the migratory-cell number increase was not statistically significant when cells were transfected with the *BTBD7*-overexpression plasmid, which was consistent with the findings of the wound healing assay (Figure 4(a-b, d-e)). The cell migration capacity was inhibited by treatment with ART, and this inhibition was reversed when cells overexpressed *BTBD7*. The expression level of EMT markers was also detected after cells were treated with ART, transfected with *BTBD7*-overexpression plasmid, or subjected to a combination of both. As shown in Figure 4(c and f), the expression of E-cadherin was decreased and that of other EMT markers was increased when NSCLC cells were treated with ART and transfected with the *BTBD7*-overexpression plasmid. These results indicate that ART inhibits A549 and H1975 cell migration by downregulating the transcription of *BTBD7*, while this inhibition is reversed when cells overexpress *BTBD7*.

## Discussion

Lung cancer is still a global health problem with highest morbidity and mortality rate among all cancers. Although conventional therapy for patients with advanced NSCLC, including chemotherapy, radiotherapy, and targeted therapy, have achieved certain beneficial effects on tumor invasion and distant metastases, their clinical efficacy is invariably limited, and the 5-year survival rate for NSCLC patients remains very low [24,25].

Metastasis is one of the leading causes of death in NSCLC patients. Although the underlying mechanism of tumor cell metastasis remains unclear, recent studies have demonstrated that tumor metastasis is closely related to EMT [26,27]. In this study, we found that ART, a main derivative of artemisinin, has an anti-migrated activity in the NSCLC cell lines, A549 and H1975. The transwell assay and wound healing assay showed that the migration capacity of these cells was inhibited significantly when cells were treated with ART for 24 h. The number of cells that migrated across the filter membrane was decreased and the relative wound width was higher for cells that were treated with ART than for control cells, and the effect of ART was found to be dose dependent. Moreover, the expression of EMT-related molecular markers in the two NSCLC cell lines was detected by western blotting. As shown in Figure 2, the protein level of E-cadherin was upregulated, while the expression of N-cadherin, vimentin, and FN1 was decreased upon treatment of cells with various concentrations of ART.

The occurrence of EMT needs specific inducers, which are divided into endogenous and exogenous. Endogenous inducers include gene mutations or overexpression and exogenous inducers include smoking, hypoxia inducible factor (HIF), and conversion growth factor-β (TGF-β), among other factors [28–30]. The abnormal expression of genes, like *p53, K-ras, c-myc*, and *RGC32*, can not only change cell morphology, but also down-regulate the expression of epithelial cell markers and up-regulate the expression of mesenchymal cell markers [31–33]. *BTBD7*, a BTB/POZ domain-containing protein, was first shown the correlation with cell proliferation and tumor formation in liver cancer [34,35]. Recent studies have reported that *BTBD7* is associated with metastasis and prognosis in primary salivary adenoid cystic carcinoma and NSCLC through regulating the protein level of E-cadherin and other markers [21,36–38]. In this study, we found ART to inhibit NSCLC cell migration by down-regulating the transcription of *BTBD7*, while this migration suppressive effect was reversed when *BTBD7* was overexpressed. The migratory-cell number counted in the transwell assay was increased and the relative wound width was decreased when cells were transfected with the *BTBD7* overexpression plasmid and co-treated with ART, which indicated the A549 and H1975 cells became more aggressive under these conditions.

## Conclusion

We demonstrated here that ART inhibits the migration capacity of the NSCLC cell lines, A549 and H1975, in a dose-dependent manner. On further investigation of the underlying mechanism, we found that the migration suppression was mediated by the down-regulation of the level of *BTBD7*, and this phenomenon was reversed by the overexpression of *BTBD7*.

## Data Availability

All data generated or analyzed during this study are included in this published article.

## Highlights

- Artesunate inhibited cell migratory in NSCLC.
- Artesunate downregulated the transcription of BTBD7.
- BTBD7 was closely connected with cell EMT in NSCLC.
